# Arterial Sleeve Lobectomy: Does Pulmonary Artery Reconstruction Type Impact Lung Function?

**DOI:** 10.3390/cancers15204971

**Published:** 2023-10-13

**Authors:** Aude Nguyen, Laurence Solovei, Charles Marty-Ané, Arnaud Bourdin, Ludovic Canaud, Pierre Alric, Kheira Hireche

**Affiliations:** 1 Department of Thoracic and Vascular Surgery, Arnaud de Villeneuve University Hospital, 191 Avenue Doyen Gaston Giraud, 34090 Montpellier, France; l-solovei@chu-montpellier.fr (L.S.); kheira.hireche@yahoo.fr (K.H.); 2 PhyMedExp, University of Montpellier, INSERM, CNRS, 34295 Montpellier, France; 3 Department of Respiratory Diseases, Arnaud de Villeneuve University Hospital, 191 Avenue Doyen Gaston Giraud, 34090 Montpellier, France

**Keywords:** non-small-cell lung cancer, pulmonary artery, sleeve lobectomy, lung perfusion, lung function

## Abstract

**Simple Summary:**

Lung cancer is the second most frequent tumor worldwide. In cases of pulmonary artery invasion, arterial sleeve lobectomy has progressively gained acceptance as an advantageous alternative to pneumonectomy, but few studies have considered lung perfusion and respiratory function. This single-center retrospective cohort study aimed to assess the impact of arterial reconstruction techniques on lung perfusion and respiratory function. Between January 2001 and December 2020, a comparative analysis of 48 patients’ preoperative and postoperative functional outcomes (FEV_1_) and 28 patients’ preoperative and postoperative lung perfusion results was conducted. Regardless of the type of vascular reconstruction, the study revealed no significant difference between the perfusion ratio of the remaining parenchyma before and after surgery. Moreover, arterial reconstruction did not negatively affect the expected postoperative respiratory function. Arterial sleeve lobectomy is a valid parenchymal-sparing technique in terms of perfusion and respiratory function.

**Abstract:**

Background: The aim of this single-center retrospective cohort study was to assess the impact of arterial reconstruction technique on lung perfusion. The second objective was to ascertain the functional validity of arterial sleeve lobectomy. Method: Between January 2001 and December 2020, a total of 81 patients underwent lobectomy with pulmonary artery (PA) reconstruction for lung cancer at the University Hospital of Montpellier. After excluding patients with an incomplete postoperative pulmonary function test, we conducted a comparative analysis of the preoperative and postoperative functional outcomes (FEV_1_) of 48 patients, as well as the preoperative and postoperative Technetium99m scintigraphic pulmonary perfusion results of 28 patients. Then, we analyzed postoperative perfusion results according to the pulmonary artery reconstruction techniques use. Results: PA reconstruction types were as follows: 9 direct angioplasties (19%), 14 patch angioplasties (29%), 7 end-to-end anastomoses (15%), 6 prosthetic bypasses (12%), 11 arterial allograft bypasses (23%), and 1 custom-made xenopericardial conduit bypass. Regardless of the type of vascular reconstruction performed, the comparative analysis of lung perfusion revealed no significant difference between the preoperative and postoperative perfusion ratio of the remaining parenchyma (median = 29.5% versus 32.5%, respectively; *p* = 0.47). Regarding the pulmonary functional test, postoperative predicted FEV_1_ significantly underestimated the actual postoperative measured FEV_1_ by about 260 mL (11.4%) of the preoperative value. The patency rate was 96% and the 5-year overall survival was 49% for a mean follow up period of 34 months. Conclusion: Lobectomy with PA reconstruction is a valid parenchymal-sparing technique in terms of perfusion and respiratory function.

## 1. Introduction

Despite the evolution of medical and multimodal treatment, non-small-cell lung cancer remains the leading cause of cancer deaths worldwide [1], with surgery being the only curative option [2]. Historically, pneumonectomy was the standard of care of central lung tumors with bronchus and/or pulmonary artery (PA) invasion. In recent years, sleeve lobectomy with PA reconstruction has progressively gained acceptance as an advantageous alternative to pneumonectomy. Initially performed on patients with impaired cardio-pulmonary reserves [3], this parenchymal-sparing technique is now performed on all patients where anatomical conditions allow complete resection [4]. Growing evidence suggests that arterial sleeve lobectomy is associated with lower postoperative complications, long term morbi-mortality [5,6,7], and better quality of life [8] when compared to pneumonectomy, even after neoadjuvant therapy [9]. However, few studies have considered the lung perfusion, respiratory function, and patency outcomes of sleeve procedures. Therefore, our aim was to investigate whether the type of arterial reconstruction has an impact on lung perfusion. This investigation involved comparing and analyzing the preoperative and postoperative perfusion of the preserved lobes using ventilation/perfusion lung scintigraphy, while considering the specific reconstruction technique employed. Our secondary aim was to evaluate postoperative lung function: we compared measured postoperative FEV_1_ (MPO FEV_1_) with predicted postoperative FEV_1_ (PPO FEV_1_).

## 2. Materials and Methods

### 2.1. Study Design and Preoperative Evaluation

We performed a retrospective analysis of the perfusion and respiratory function data of patients who underwent sleeve resection for lung tumors with PA invasion, requiring partial or complete arterial reconstruction at Montpellier University Hospital, between January 2001 and December 2020. Complete preoperative and postoperative functional results were available for 48 patients, of which complete preoperative and postoperative perfusion data were available for 28. We excluded patients having undergone only bronchial sleeve resection, patients with incomplete preoperative and postoperative pulmonary function tests (PFTs), and patients with tumors other than NSCLC. Preoperative assessment included a contrast-enhanced thoracic–abdominal and pelvic computed tomography, brain imaging, and whole-body fluorodeoxyglucose positron emission tomography (PET/CTFDG), the latter having replaced bone scintigraphy since 2007. Preoperative pulmonary function studies were performed on all patients to assess first-second forced expiratory volume (FEV_1_) and diffusing capacity of the lung for carbon monoxide (DLCO). Direct measurement of whole-body maximal oxygen consumption (VO2-max test) and TC 99mperfusion lung scintigraphy were performed when needed according to European guidelines [10]. PPO FEV_1_ was estimated using the formula described by Brunelli: [11]

Predicted postoperative FEV1 = Preoperative FEV1 × (1 − number of functional segments resected/number of total functional segments).

Tumor stage was determined according to the eighth edition TNM Staging classification [12]. Invasive nodal staging using endobronchial ultrasound biopsy or mediastinoscopy was performed in cases of N2 clinical stages with mediastinal uptake on PET/CTFDG. All patients with clinical stage IIIA-N2 and higher received neoadjuvant therapy mediastinal restaging after induction therapy and prior to resection. Data were obtained from patient case notes and electronic patient records, then entered into a dedicated anonymized database (Excel, Microsoft Corp, Redmond, WA, USA). The study was approved by the local ethics committee and Institutional Review Board (N°IRB-MTP_2021_07_202100888) with a waiver of individual patient consent.

### 2.2. Surgical Procedure

Four experienced surgeons were in charge of the patients. The cause of arterial invasion was either hilar tumors or lymph node disease. The procedure was performed under general anesthesia with double-lumen endotracheal intubation. Lung lobectomy with radical lymph node dissection was performed in all patients via a posterolateral thoracotomy. Oncologic and technical feasibility of the arterial sleeve lobectomy was assessed by the surgeon after proximal and distal control of the PA. Systemic heparin sodium (0.5 mg·kg^−1^) was intravenously administered. Then, vascular clamps were placed at the proximal and distal disease-free PA segment. The tumor was resected en bloc with the involved portion of the PA and other adjacent structures if required. All resection margins were ascertained by frozen section analysis. The modality of PA reconstruction was assessed after resection. Thus, we considered 4 types of PA reconstruction: direct angioplasty, patch angioplasty using either prosthetic (HemaCarotid patch, Maquet^®^ Gentige Group, Rastatt, Germany) or biological bovine pericardium patches (XenoSure Biological Patch; LeMaitre Vascular, Burlington, MA, USA), end-to-end anastomosis, and bypass using prosthetic conduits (GORE-TEX^®^, WL Gore&Associates, Flagstaff, AZ, USA) or cryopreserved allograft (PA or thoracic aorta) in cases of extensive PA involvement precluding direct reconstruction. Vascular anastomoses were performed with 5-0 or 6-0 polypropylene thread. At the end of the vascular procedure, the distal clamp was removed before tying the suture in order to purge and to check the congruence of the reconstruction (absence of kinking and twisting). When a double sleeve was needed, the bronchial anastomosis was performed first, through separate PDS 4.0 stitches. All patients received 6000 UI subcutaneous low-molecular-weight heparin for 15 days after surgery. [13]

### 2.3. Follow Up

Postoperative follow up data were collected from several sources: consultation and hospitalization reports and personal contact with patients and/or their families. The national death registry was also consulted. We collected the most recent PFT for all patients. When pre- and postoperative lung scintigraphy results were available, perfusion and ventilation assessments were recorded for each lobe. Patency PA reconstruction was assessed on the most recent contrast-enhanced thoracic CT.

### 2.4. Statistical Analysis

Statistical analyses were performed using RStudio version 4.0.1. (R Foundation, Vienna, Austria) and ExcelStat (Microsoft Corp, Redmond, WA, USA).

Scintigraphic assessments of lung perfusion were compared using the Wilcoxon signed-rank test for paired data. We additionally performed a subgroup analysis according to the type of PA reconstruction technique. The difference between predicted and measured postoperative FEV_1_ is represented by ∆FEV_1_ (= MPO FEV_1_ − PPO FEV_1_). Comparison of MPO FEV_1_ and PPO FEV_1_ was performed using a linear regression model. Univariate and multivariate analysis was also performed to identify confounding factors of lower FEV_1_. The Kaplan–Meier method was used to perform survival analysis. A *p*-value less than 0.05 was considered significant, without correction for multiple statistical tests.

## 3. Results

### 3.1. Patient Characteristics and Overall Survival

Patient characteristics and surgical details are described in Table 1. 69% were men with a median age at surgery of 67 years. Left upper lobectomy was the most common surgical procedure (68%), and squamous cell carcinoma was the predominant pathological subtype. The types of PA reconstruction included 9 direct angioplasties (19%), 14 patch angioplasties (29%), 7 end-to-end anastomoses (15%) and 18 bypass reconstructions (37%). Seventeen patients (35%) had double arterial and bronchial sleeve lobectomy. The median follow up period was 34 months (0.5–153 months). The 5-year overall survival was 49%. Lung scintigraphy results were available for 28 patients.

### 3.2. Patency and Perfusion Results

**Figure 1:**
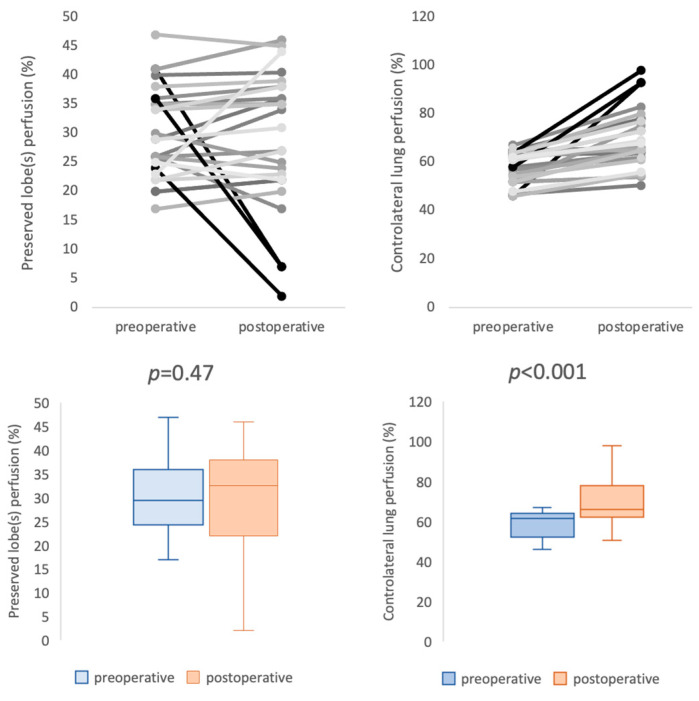
Results of preoperative and postoperative lung perfusion. It reveals three very low values (in bold) of postoperative perfusion corresponding to the cases of complete or partial thrombosis.

**Figure 2:**
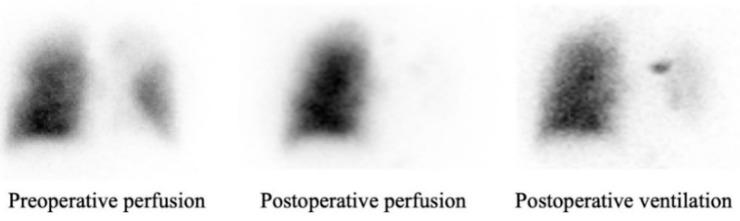
Complete thrombosis of prosthetic bypass on ventilation/perfusion lung scintigraphy.

**Figure 3:**
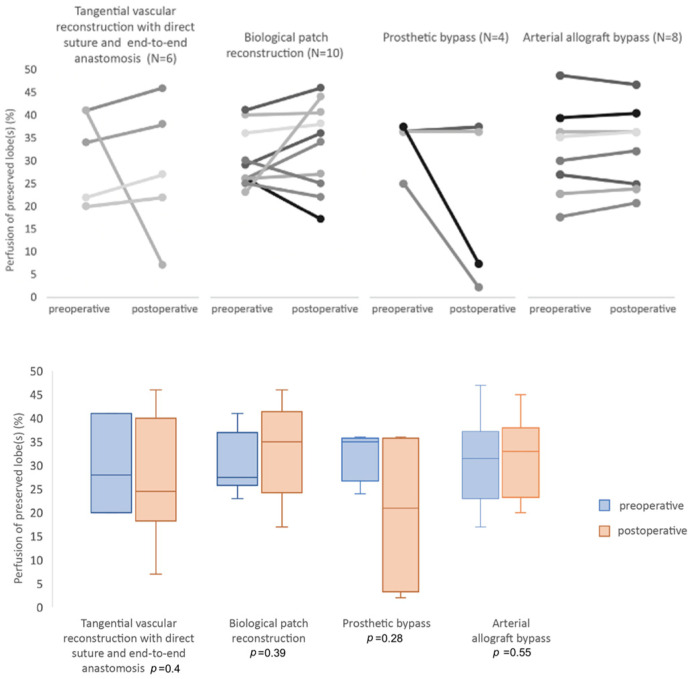
Preoperative and postoperative perfusion of preserved lobe according to the technique of reconstruction.

CT scan follow up was performed 6 to 24 months after surgery (mean 14 months). Patency rate was 96%. Three patients (6.3%) had a complete or partial PA thrombosis. For these patients, the type of PA reconstruction was prosthetic bypass in the left upper lobectomy. Twenty-eight patients had complete data on preoperative and postoperative ventilation/perfusion lung scintigraphy. Median time between surgery and postoperative lung scintigraphy was 32 months (13–48 months). For those patients, the type of PA reconstructions used were 3 direct angioplasties (11%), 3 end-to-end anastomosis (11%), 10 patch angioplasties (36%), 4 prosthetic bypasses (14%), 7 cryopreserved arterial allograft bypasses (25%), and 1 custom-made xenopericardial patch bypass (3%). There was no significant difference between the preoperative and postoperative perfusion ratio of the remaining parenchyma on the operated side (29.5% (25–36) vs. 32.5% (22–38), *p* = 0.47). However, the postoperative perfusion of the contralateral lung increased significantly (61.5% (52–64) vs. 66% (62–77), *p* < 0.001) (Figure 1), while the lowest perfusion values were observed in patients with partial or complete thrombosis (*n* = 3). No perfusion defects were observed on lung scintigraphy, except in one patient with complete prosthetic bypass thrombosis (Figure 2). Perfusion analysis according to the type of reconstruction did not show any statistically significant difference for each type of arterial reconstruction (Figure 3).

### 3.3. Functional Results

PFTs were performed 7 to 26 months after surgery (median 12 months). Table 1 shows the preoperative and postoperative pulmonary function tests. FEV_1_ was significatively reduced by 400 mL (15% of the preoperative value, *p* < 0.001). The preoperative and postoperative DLCO values were 63% (53–79) and 52 % (62–71) (*p* < 0.01), respectively, also showing significant loss.

It should be noted that the comparison of pre- and postoperative FEV_1_ in the 17 patients who underwent a double sleeve procedure yielded non-significant results.

**Figure 4:**
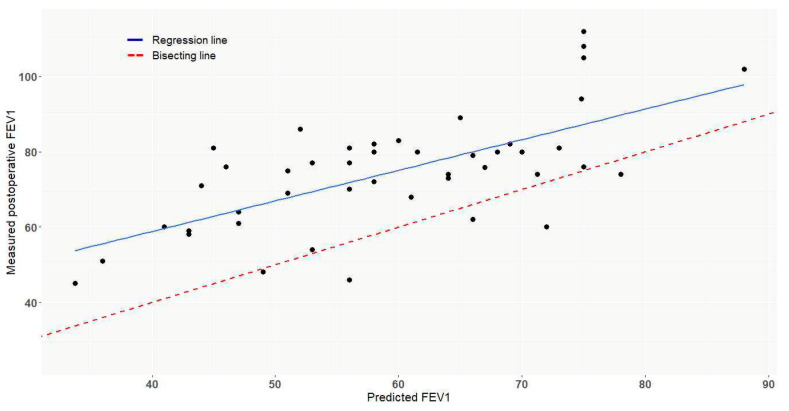
Correlation between measured postoperative FEV_1_ (%) and predicted postoperative FEV_1_ (%) (r = 0.006, *p* < 0.001).

Median MPO FEV_1_ was 75 % (63–81%). There was a statistically significant linear correlation between MPO and PPO FEV_1_ (r = 0.66, *p* < 0.001) (Figure 4). However, median MPO FEV_1_ was 260 mL (11.4% of the preoperative value) higher than PPO FEV_1_. In univariate analysis, PA reconstruction technique did not affect ∆FEV_1._ However, multivariate analysis revealed prosthetic bypass to be a predictive factor of worse ∆FEV_1_ (*p* = 0.03) (Table 2).

## 4. Discussion

In this study, we report further evidence regarding the functional validity of PA reconstruction during parenchymal-sparing lung cancer surgery whatever the type of arterial reconstruction. Arterial sleeve lobectomy has been widely accepted in our center since the early 2000s, with the combination of vascular and thoracic surgery in the same department being a determining factor in the development of this complex procedure in routine practice [14,15].

The incidence of arterial sleeve resection remains low, with reported rates ranging from 1.5% to 3.7% [16,17], which are generally related to the more technically demanding procedure rather than to bronchial sleeve lobectomy and pneumonectomy. The choice of reconstruction techniques and materials remains a major issue. While several descriptions of the different options exist, no definite recommendations on this subject have been made [18,19,20].

It should also be highlighted that even the definition of sleeve lobectomy remains fuzzy. According to Vannucci et al. [19], “PA reconstruction could be defined as any kind of arterial wall resection, repaired by a hand suture requiring a proximal clamp, placed after systemic heparinization”, whereas some surgeons may consider tangential excisions of the arterial wall with primary closure as part of an anatomic pulmonary resection for lung cancer. However, previous studies reported high rates of thrombosis and pulmonary embolism when performing tangential primary repair of the PA. In our series, none of the nine patients who underwent direct angioplasty developed perfusion defects or pulmonary embolism.

Despite the growing acceptance and the confirmed safety and oncologic reliability of this reconstructive procedure [5,16], only two studies have paid attention to the impact of PA reconstruction on postoperative pulmonary perfusion. The first postoperative perfusion data were reported by Rendina et al. in 1999 [21], and demonstrated normal perfusion of the preserved lobe of 24–42% of total lung perfusion. However, this study included only three cases (5.8%) of PA replacement. In 2009, Schirren et al. [22] conducted a prospective study of 100 consecutive patients undergoing bronchial or bronchovascular sleeve resection. The authors reported 26% and 33% lung perfusion of the residual lobe on the left and the right side, respectively, and showed that the proportion of perfusion of the preserved lobe gradually improved and stabilized at 6 months. However, the authors did not provide details regarding the type of PA reconstruction. With a median postoperative perfusion of 32.5% (22–38%) and median time from surgery to postoperative scintigraphy of 32 months, our findings are in line with the latest reports [21,22]. Moreover, our study is the first to provide pre- and postoperative comparative results of the preserved parenchyma perfusion according to the type of PA reconstruction, including 18 PA replacements (38%). Subgroup analysis did not show any perfusion difference for each type of PA reconstruction. However, it should be noted that three cases of thrombosis occurred in prosthetic bypass grafts, resulting in a major defect on postoperative lung scintigraphy. This finding supports previous observations advocating the use of biological materials in order to decrease thrombotic risk [23,24,25,26]. The type of substitute is not the only factor of perfusion defects; PA bypass repositioning during pulmonary re-expansion can be a source of twist or kink and must be carefully assessed. Also, PA resection without combined bronchial sleeve resection may result in thrombosis by excessive tensing of an end-to-end anastomosis. Considering the encouraging perfusion results over a median follow up of 34 months, we believe that bypass reconstruction should be considered whenever uncertainty persists as to unsatisfactory PA reconstruction by a tensioned end-to-end anastomosis.

The results of our study show that arterial reconstruction does not negatively affect the expected postoperative FEV_1_ value. By comparing FEV_1_ PPO and FEV_1_ MPO, we considered the volume of lung resected. Furthermore, we found that PPO FEV_1_ significantly underestimates MPO FEV_1_ by about 260 mL (11.4% of the preoperative value). This underestimation should be factored in when offering lung resection to patients who might be excluded from any surgical program because of impaired preoperative lung functions. Multivariate analysis revealed that prosthetic bypass was a significant factor associated with lower MPO FEV_1_. One might wonder about the relationship between arterial reconstruction and respiratory function; we answer that the understanding of hypoxic vasoconstriction and bronchoconstriction, especially in COPD patients, along with the disparities in viscoelastic properties between native tissue and vascular substitutes, has raised questions regarding the potential impact of arterial clamping and the patency of arterial reconstruction on postoperative respiratory function following such surgical procedures. Furthermore, the direct influence of pulmonary embolism on bronchoconstriction, increased airway resistance, and decreased pulmonary compliance has been well established [27]. In 2017, Danielsbacka and colleagues [28] demonstrated that patients with acute pulmonary embolism experience reduced lung function and functional capacity, highlighting how a perfusion defect can impair respiratory function. Consistent with Danielsbacka’s findings, postoperative lung scintigraphy in the three cases of arterial thromboses reported in this study revealed that the perfusion defect closely resembled the ventilation defect (Figure 2).

Previous studies have examined the permanent PFT loss following lung resection and reported that the percentage differences in FEV_1_ and DLCO at 6 to 12 months after standard lobectomy compared with the preoperative values were to be 8.8% to 17.6% and 3.6 to 11.5%, respectively [29,30,31,32,33]. The historical series by Deslauriers et al. [34] evaluating the long-term cardiopulmonary function in 100 pneumonectomy patients reported a worsening of FEV_1_ by approximately 30% and a reduction of DLCO by 33%. In the present study, the losses of FEV_1_ and DLCO were similar to those of previous reports on PFT following standard lobectomy and significantly less in comparison with that after pneumonectomy.

## 5. Limitations

First, this study is limited by a selection bias related to the retrospective design. Second, the small number of patients who had pre- and postoperative scintigraphic evaluation drastically reduced the verification effectiveness of our study. Third, because CT scanning is not a dynamic examination, a more accurate analysis of the position and angulation of the PA reconstruction during respiratory movements could not be performed. Fourth, there is an obvious bias related to the disparity in the postoperative evaluation time among patients. In future, prospective studies which employ systematic pre- and postoperative perfusion scans and dynamic MRI would allow a precise analysis of the perfusion results according to the congruence of the PA reconstruction.

## 6. Conclusions

Lobectomy with arterial reconstruction is a valid parenchymal-sparing technique in terms of perfusion and respiratory function. Priority must be given to the use of biological materials for arterial reconstruction.

## Figures and Tables

**Table 1:** Patient characteristics and surgical details.

Characteristics		
Gender (n/%)		
Male	33	69%
Female	15	31%
Age (median/IQR)	67 (55–71)	
Smoking status (n/%)		
Never smoked	4	8%
Past smoker	38	79%
Current smoker	6	13%
Pack-year > 30	29	60%
IMC (Mean, kg·m^−1^)	24	
Comorbidity		
Cancer	10	21%
Diabetes	4	8%
COPD	12	25%
Cardiac disease	13	27%
Pulmonary function test		
Preoperative FEV1 (L, %)	2.2 (1.8–2.6)	80% (69–89)
Measured postoperative FEV1 (L, %)	1.9 (1.6–2.3)	75% (63–81)
Predicted postoperative FEV1 (L, %)	1.1 (0.95–2.6)	58% (50–69)
Preoperative DLCO (mL/mmHg/Mi, %)	14.5 (12.1–17.8)	63% (53–79)
Postoperative DLCO (mL/mmHg/Mi, %)	14.6 (11.9–17.4)	52% (62–71)
Neoadjuvant therapy (n/%)	16	33%
Adjuvant therapy (n/%)	27	56%
Surgical resection (n/%)		
Right upper lobectomy	6	13%
Middle lobectomy	1	2%
Upper bilobectomy	2	4%
Left upper lobectomy	34	71%
Left lower lobectomy	5	10%
Histology (n/%)		
Squamous cell carcinoma	27	56%
Adenocarcinoma	18	38%
Neuroendocrine carcinoma	2	4%
Undifferentiated carcinoma	1	2%
cT status (n/%)		
T1	6	13%
T2	15	31%
T3	13	27%
T4	11	23%
Unknown	3	6%
cN status (n/%)		
N0	21	44%
N1	14	29%
N2	10	21%
Unknown	3	6%
pT status (n/%)		
T1	6	13%
T2	12	25%
T3	19	40%
T4	7	14%
Unknown	4	8%
pN status (n/%)		
N0	19	40%
N1	24	50%
N2	3	6%
Unknown	2	4%
Double sleeve (n/%)	17	35%
PA reconstruction technique (n/%)		
Direct angioplasty	9	19%
Patch angioplasty	14	29%
Biologic	13	27%
Prosthetic	1	2%
End-to-end anastomosis	7	15%
Bypass	18	37%
Prosthetic	6	12%
Cryopreserved arterial allograft	11	23%
Custom-made xenopericardial conduit	1	2%

**Table 2:** Univariate and multivariate analysis of ∆FEV_1._ Multivariate analysis revealed prosthetic bypass to be a predictive factor of worse ∆FEV_1_ (*p* = 0.03).

Variable	Number of Patients (*n* = 45)	*p*-Value in Univariate Analysis	*p*-Value in Multivariate Analysis
Direct angioplasty	9	0.43	0.09
End-to-end anastomosis	7	0.16	0.07
Biological patch angioplasty	13	0.28	0.12
Prosthetic bypass	6	0.06	0.03
Arterial allograft bypass	11	0.42	0.10

## Data Availability

The data that support the findings of this study are available from the corresponding author upon reasonable request.

## Abbreviations

PA	Pulmonary artery
FEV_1_	First-second forced expiratory volume
MPO FEV_1_	Measured postoperative FEV_1_
PPO FEV_1_	Predicted postoperative FEV_1_
PFT	Pulmonary function test
PET/CTFDG	Fluorodeoxyglucose positron emission tomography
DLCO	Diffusing capacity of the lung for carbon monoxide
